# Dye-sensitized Pt@TiO_2_ core–shell nanostructures for the efficient photocatalytic generation of hydrogen

**DOI:** 10.3762/bjnano.5.41

**Published:** 2014-03-26

**Authors:** Jun Fang, Lisha Yin, Shaowen Cao, Yusen Liao, Can Xue

**Affiliations:** 1 Solar Fuels Laboratory, School of Materials Science and Engineering, Nanyang Technological University, 50 Nanyang Avenue, Singapore 639798, Singapore

**Keywords:** charge transfer, dye-sensitization, photocatalysis, photocatalyst, solar fuels, water splitting

## Abstract

Pt@TiO_2_ core–shell nanostructures were prepared through a hydrothermal method. The dye-sensitization of these Pt@TiO_2_ core–shell structures allows for a high photocatalytic activity for the generation of hydrogen from proton reduction under visible-light irradiation. When the dyes and TiO_2_ were co-excited through the combination of two irradiation beams with different wavelengths, a synergic effect was observed, which led to a greatly enhanced H_2_ generation yield. This is attributed to the rational spatial distribution of the three components (dye, TiO_2_, Pt), and the vectored transport of photogenerated electrons from the dye to the Pt particles via the TiO_2_ particle bridge.

## Introduction

Since Honda and Fujishima reported the effective hydrogen evolution from water splitting by a TiO_2_ and Pt electrode in a photoelectrochemical cell in the early 1970s [1], TiO_2_ has received extensive attention as one of the promising semiconductor photocatalysts, because of its high chemical stability, low cost and non-toxicity [2–5]. However, it suffers from the wide band gap (3.2–3.4 eV), which restricts the utilization of visible light, and the high recombination rate of photogenerated electrons and holes often leads to low quantum yields and a poor photocatalytic activity [6]. Tremendous efforts have been made to improve the photocatalytic performance of TiO_2_. One typical strategy is prolonging the lifetime of the electron–hole pair through deposition of noble metal (e.g., Pt) nanoparticles as co-catalysts that can act as electron-sinks to achieve effective charge separation on TiO_2_ [7–11]. Dye-sensitization has been widely used to enable visible light harvesting by wide band gap semiconductors. Since the seminal work reported by O’Regan and Grätzel in 1991 [12], various types of dyes have been explored, and some of them allow for the reduction of protons into hydrogen gas through visible-light-driven photocatalytic processes [13–17]. Herein, we use erythrosin B (ErB) sensitized Pt@TiO_2_ core–shell nanoparticles for the highly-efficient photocatalytic generation of hydrogen under visible-light irradiation. In this rational design of the ternary structure, the TiO_2_ particle acts as a bridge that allows for the effective electron transfer pathway of excited ErB→TiO_2_→Pt. Importantly, we found that when the TiO_2_ bridges are excited simultaneously, the dye-sensitization-driven H_2_ evolution showed a much higher efficiency as compared to the situation with no excitation of TiO_2_. This kind of synergic effect reveals a new direction for improving the efficiency of composite photocatalysts by using selective excitation wavelengths.

## Results and Discussion

**Figure 1:**
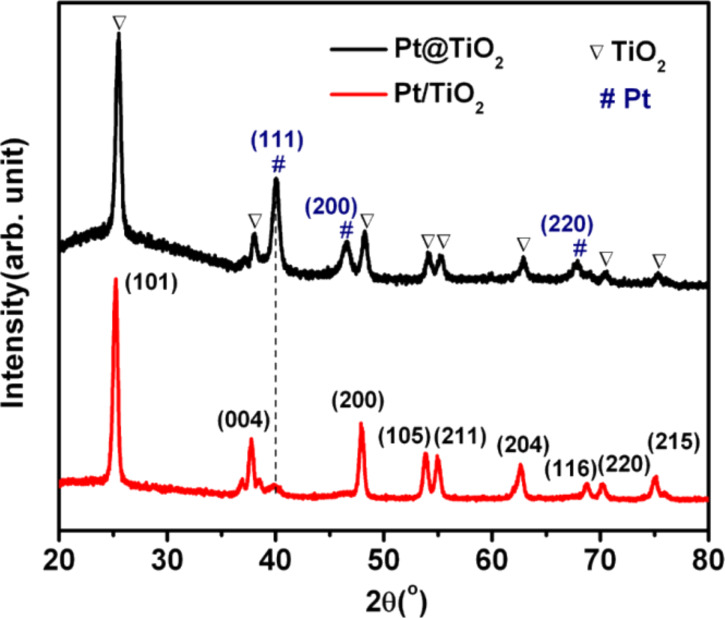
XRD patterns of Pt@TiO_2_ and Pt/TiO_2_ samples.

The Pt@TiO_2_ core–shell nanoparticles were prepared trough a hydrothermal process by using Pt nanoparticles and TiF_4_ as the precursor. The crystalline structure was determined by XRD, as shown in Figure 1. After the hydrothermal reaction, the TiO_2_ was transformed into anatase phase, which could be well indexed to the standard anatase TiO_2_ (JCPDS Card No. 83-2243). The three additional diffraction peaks shown in Figure 1 could be assigned to the face-centered metallic Pt phase, with the positions at 40.0^o^, 46.6^o^ and 67.9^o^ representing the spacing of the (111), (200) and (220) planes, respectively. This indicates the retaining of the Pt nanoparticle cores after the hydrothermal reaction. As a control sample, Pt/TiO_2_ was prepared through the photodeposition of Pt (1% in mole fraction) onto pure TiO_2_ particles that were prepared through the same hydrothermal method without using Pt nanoparticles. Due to the low Pt loading, we could only observe a weak diffraction peak at 40^o^ corresponding to the Pt (111) lattice spacing, which could indicate successful loading of metallic Pt nanoparticles onto TiO_2_ particles. The molar ratio of Pt to Ti was estimated to be about 6.7% according to EDX analysis (Figure S1, Supporting Information File 1).

**Figure 2:**
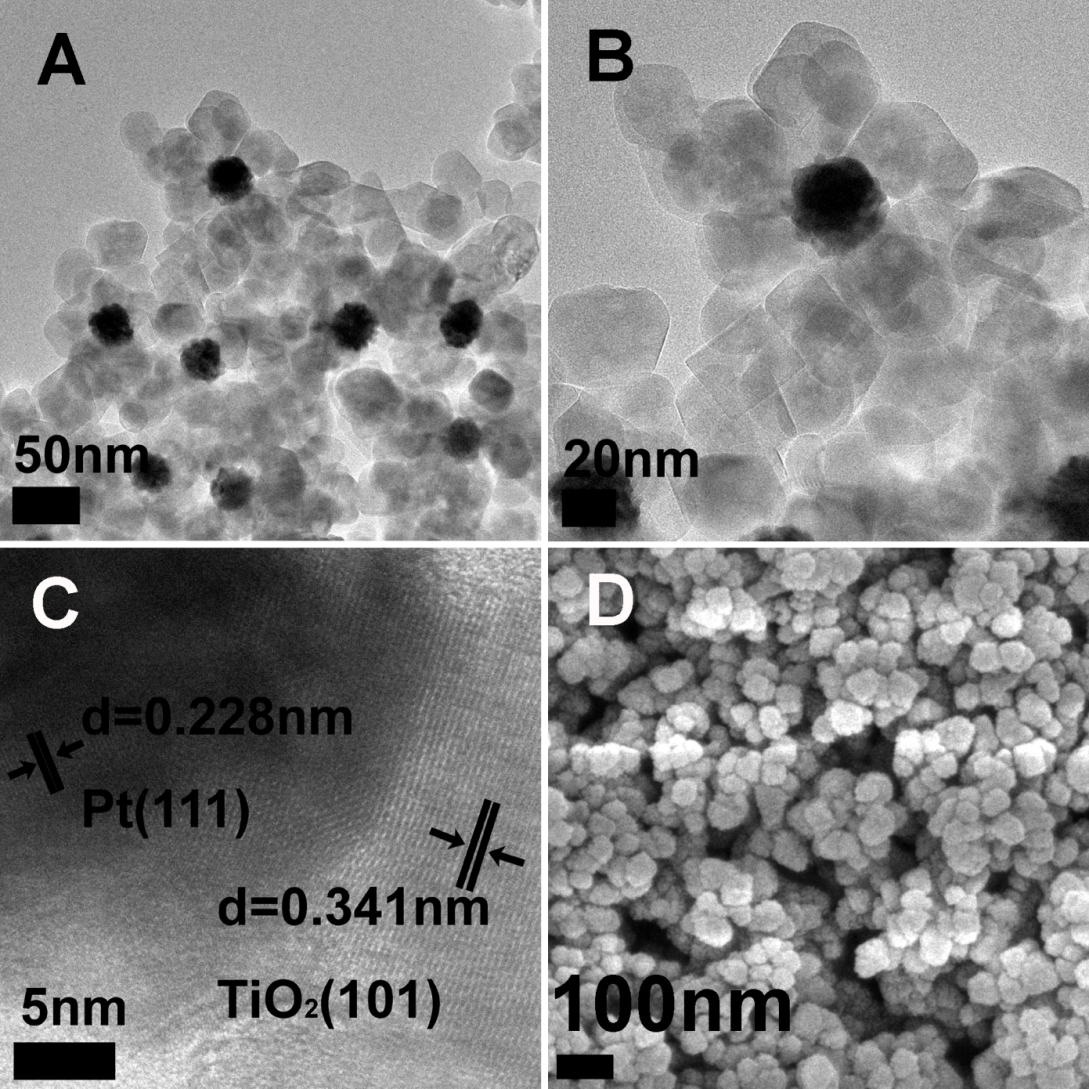
TEM and SEM images of the Pt@TiO_2_ sample. (A) (B) TEM images of Pt@TiO_2_, (C) HRTEM images of Pt@TiO_2_, (D) SEM image of Pt@TiO_2_.

The core–shell morphology of the prepared Pt@TiO_2_ nanostructures was confirmed by TEM and SEM examination. As shown in Figure 2A and 2B, the core–shell particles appear as flower-like structures, in which the Pt nanoparticles as the cores show an average diameter of 30 nm, and the TiO_2_ shell thickness is around 60 nm. The HRTEM image (Figure 2C) indicates lattice distances of 0.228 nm and 0.341 nm, which correspond to the (111) spacing of the core Pt particle and the (101) spacing of the anatase TiO_2_ shell. The SEM image (Figure 2D) reveals that these core–shell Pt@TiO2 structures appear like a large particle with scraggy surfaces and an average diameter of about 150 nm. These observations confirm that all Pt nanoparticles are well encapsulated by TiO_2_ shells. Nevertheless, we note that the TiO_2_ shell does not compactly cover all Pt surfaces, which allows for the proton reduction and H_2_ evolution on the uncovered surface area of Pt. For the control sample Pt/TiO_2_, the TiO_2_ particles were synthesized through a hydrothermal method that was followed by the photodeposition of Pt nanoparticles, as shown in Figure S2 (Supporting Information File 1). The TiO_2_ particles are in a solid spherical shape and composed by nanoparticle aggregation, and the average diameter of deposited Pt nanoparticles is about 5 nm.

**Figure 3:**
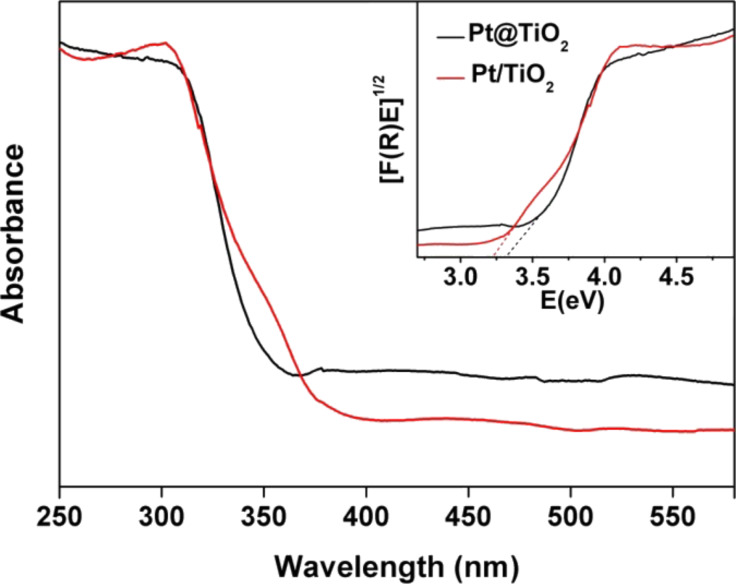
UV–vis diffuse reflectance spectra of the Pt@TiO_2_ and Pt/TiO_2_ samples.

Figure 3 shows the UV–vis diffuse reflectance spectra of the Pt@TiO_2_ core–shell nanostructures and the Pt/TiO_2_ control sample. The absorption from 250 to ca. 380 nm can be attributed to the band edge absorption of anatase TiO_2_. The band gaps of both samples are calculated according to the modified Kubelka–Munk function 
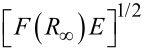
 vs the energy the of absorbed light, *E* [18]. And the plots shown in the inset of Figure 3 reveal the band gap value as 3.3 eV for Pt@TiO_2_ and 3.2 eV for Pt/TiO_2_, which indicates that the combination of Pt nanoparticles with TiO_2_ did not significantly influence the band gap energy of the TiO_2_ component.

The photocatalytic tests were carried out by suspending the Pt@TiO_2_ sample (5 mg) in an aqueous solution containing 0.2 wt % ErB and 15 wt % triethanolamine (TEOA) that acts as the electron donor. In this study, we employed two different irradiation wavelengths for the purpose of separating the excitation of TiO_2_ and ErB, which will help us to explore the potential synergic effect between the two excitations. The primary irradiation with a wavelength of 550 ± 20 nm (light A) is to excite ErB since the main absorbance peak of ErB is located at about 550 nm. A secondary irradiation with a wavelength of 400 ± 10 nm (light B) is to excite the defect/impurity states of TiO_2_, while ErB exhibit a minimum absorption in this range.

**Figure 4:**
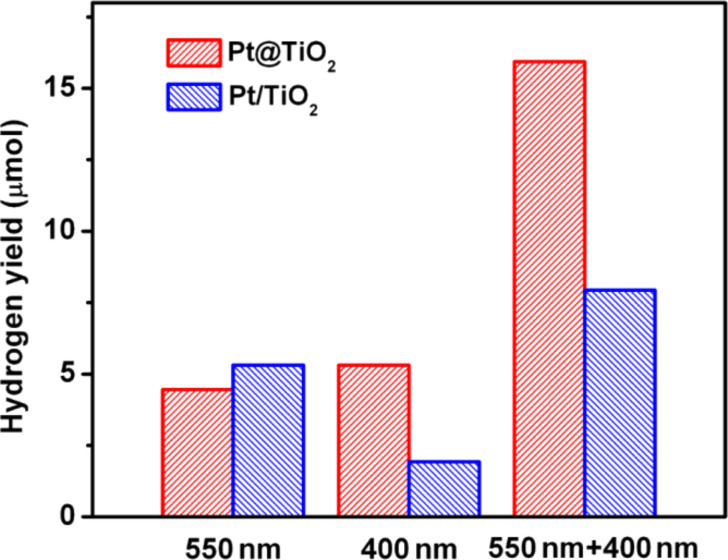
The H_2_ yield from Pt@TiO_2_ and Pt/TiO_2_ for water splitting under irridiation with the given for 2 h while using TEOA as the sacrificial reagent.

As shown in Figure 4, after the individual irradiation with light A (550 ± 20 nm) or light B (400 ± 10 nm) for 2 h, the ErB-sensitized Pt@TiO_2_ core–shell structure showed generated H_2_ amounts of 4.5 μmol and 5.3 μmol, respectively. However, when light A and light B are used simultaneously, to our surprise, the 2 h irradiation led to a H_2_ amount of 15.9 μmol, which is significantly higher than the sum of the two generated H_2_ amounts under individual irradiation of light A and B (9.8 μmol). This observation suggests that in the ErB-sensitized Pt@TiO_2_ core–shell structure, a synergic effect exists between the excitation of ErB and TiO_2_, which plays an important role in the photocatalytic hydrogen generation.

**Figure 5:**
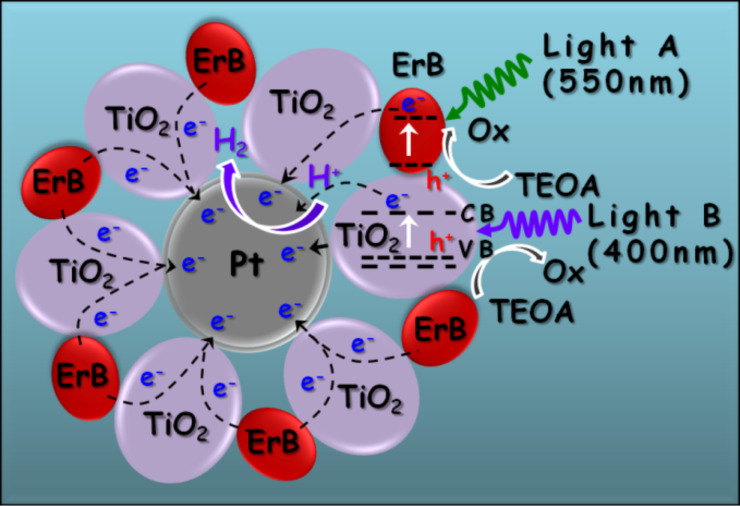
Schematic illustration of the photocatalytic H_2_ generation by ErB-sensitized Pt@TiO_2_ core–shell nanostructures under irradiation of light A (550 ± 20 nm) and/or light B (400 ± 10 nm).

The observed synergic effect could be attributed to the electron transport in TiO_2_ particles. Since the dye-sensitization induces an electron transfer from the excited ErB to TiO_2_, these electrons have to be transported through the TiO_2_ particles with a maximum distance of ca. 60 nm to reach the Pt surface for the reduction of protons to H_2_. When TiO_2_ is simultaneously excited by the 400 ± 10 nm light, though it is weak, the charge carrier concentration in TiO_2_ becomes higher, which increases the conductivity of TiO_2_. Thereby, the vectored electron transfer from ErB to the core Pt particle via TiO_2_ bridges becomes more effective, which leading to enhanced yield of H_2_. The principle is depicted in Figure 5, and the energy diagram is shown in Figure S3 (Supporting Information File 1).

In comparison, under individual irradiation of light A or light B for 2 h, the Pt/TiO_2_ sample showed a H_2_ generation of 5.3 μmol and 1.9 μmol, respectively. While the simultaneous irradiation with light A and B for 2 h led to the generation of 7.9 μmol H_2_, which is only slightly higher than the sum of the H_2_ generation amount under individual irradiation of light A and B (7.2 μmol). This indicates that the synergic effect may also exist in the Pt-loaded TiO_2_ particles, but with a less significant role as compared to the Pt@TiO_2_ core–shell structure. This may be because the post-loaded Pt nanoparticles are randomly distributed on the outer surfaces of TiO_2_ particles, thus the electron transfer path from ErB to Pt becomes less oriented through TiO_2_. In addition, the Pt nanoparticles would occupy some reactive sites on the TiO_2_ surface, which also reduces the efficiency of the dye-sensitization.

## Conclusion

We have prepared Pt@TiO_2_ core–shell nanostructures through a one-step hydrothermal method. Upon ErB sensitization, the Pt@TiO_2_ core–shell photocatalysts exhibit high visible-light activity for the generation of H_2_ from proton reduction. Significantly, we observed a synergic effect that allows for a greatly enhanced activity for the H_2_ generation when the ErB and TiO_2_ are co-excited through the combination of two irradiation beams at different wavelengths. The enhancement is attributed to the rational spatial distribution of three components (ErB, TiO_2_, Pt), and the vectored transport of photogenerated electrons from ErB to Pt particles via the TiO_2_ particle bridge. The presented core–shell structures and the observed synergic effect would provide a new direction for improving the efficiency of composite photocatalysts by using selective excitation wavelengths.

## Experimental

**Seed growth of 30 nm Pt nanoparticles:** Seed Pt nanoparticles were prepared first. Typically, an aqueous solution of H_2_PtCl_6_ (3.8 mM, 7 mL) was added into 90 mL deionized (DI) water and heated to boil under stirring. After that, 2.2 mL aqueous solution containing 1% trisodium citrate and 0.05% citrate acid was added, followed by a quick injection of freshly prepared NaBH_4_ solution (0.08%, 1.1 mL). After 10 min, the solution was cooled down to room temperature, and used as the solution of seed nanoparticles (≈4 nm). For further growth into 16 nm Pt nanoparticles, 1 mL of this Pt seed solution was added into 29 mL DI water, followed by the addition of 0.09 mL solution of H_2_PtCl_6_ (0.2 M) and 0.5 mL of a solution containing 1% sodium citrate and 1.25% L-ascorbic acid. The solution was kept under stirring and heated to boil. After 30 min, the solution was cooled down to the room temperature and used as the 16 nm Pt seed solution for further growth into 30 nm Pt nanoparticles. In a typical run, 4 mL of the 16 nm Pt particle solution was mixed with 26 mL DI water. Then 0.09 mL solution of H_2_PtCl_6_ (0.2 M) was added, followed by addition of 0.5 mL solution containing 1% trisodium citrate and 1.25% L-ascorbic acid. The solution was kept stirring and heated to boil, and after 30 min of boiling, the solution was cooled down to room temperature and used the solution of 30 nm Pt nanoparticles.

**Preparation of Pt@TiO_2_ core–shell nanostructures:** The Pt@TiO_2_ core–shell nanostructures were synthesized through a hydrothermal method. Typically, 15 mL solution of the as-prepared 30 nm Pt nanoparticles was mixed with 4.5 mL aqueous solution of TiF_4_ (0.04 M). The mixture was kept stirring for 10 min, diluted into 80 mL DI water, and then transferred into a 100 mL Teflon-lined stainless steel autoclave, which was treated at 180 °C for 24 h. After that, the product was cooled down to room temperature, centrifuged and washed with deionized water for three times, and dried in a vacuum oven.

**Characterizations:** The crystalline phases of the samples were examined by powder X-ray diffraction (XRD) on a Shimazu XRD-6000 X-ray diffractometer (Cu Kα radiation) with a scanning speed of 2°/min in the 2θ range from 20 to 80°. Diffuse reflectance UV–vis spectra were acquired on a Lambda 750 UV–vis–NIR spectrophotometer (Perkin Elmer, USA). The morphology of the Pt@TiO_2_ nanocomposites were investigated by field emission scanning electron microscopy (SEM, JEOL JSM-7600F) with energy-dispersive X-ray analysis system and transmission electron microscopy (TEM, JEOL JEM-2100) at an accelerating voltage at 200 kV.

**Photocatalytic generation of H_2_ with erythrosin B-sensitized Pt@TiO_2_ core–shell particles:** In a typical run, 5 mg Pt@TiO_2_ photocatalyst was dispersed into 10 mL of an aqueous solution containing triethanolamine (TEOA, 15 wt %) as electron donor and erythrosin B (0.2 wt %) as the photo-sensitizing dye. The suspension was sealed in a quartz vessel and purged with Argon for 30 min to remove the residual oxygen. After that, the vessel was exposed under a 300 W Xenon lamp (MAX-302, Asahi Spectra Co. Ltd.) coupled with a band pass filter (λ = 400 ± 10 nm or 550 ± 20 nm) to evaluate the photocatalytic H_2_ generation yield. The gas products were analyzed periodically by an Agilent 7890A gas chromatograph (GC) with a thermal conductivity detector (TCD).

## Supporting Information

File 1 Additional experimental data.
