# Supplementary material for: Dye-sensitized Pt@TiO2 core–shell nanostructures for the efficient photocatalytic generation of hydrogen
Source: Beilstein J Nanotechnol. 2014 Mar 26;5:360–4. doi: 10.3762/bjnano.5.41 (PMC3999845; doi:10.3762/bjnano.5.41)
Supplement: File 1 — Additional experimental data. [file Beilstein_J_Nanotechnol-05-360-s001.pdf]

Supporting Information

for

Dye-sensitized Pt@TiO_2 core–shell nanostructures for the efficient photocatalytic generation of hydrogen

Jun Fang[1], Lisha Yin[1], Shaowen Cao[1], Yusen Liao[1] and Can Xue[1,]*

Address: [1]Solar Fuels Laboratory, School of Materials Science and Engineering, Nanyang Technological University, 50 Nanyang Avenue, Singapore 639798, Singapore

Email: Can Xue - cxue@ntu.edu.sg

* Corresponding author

Additional experimental data

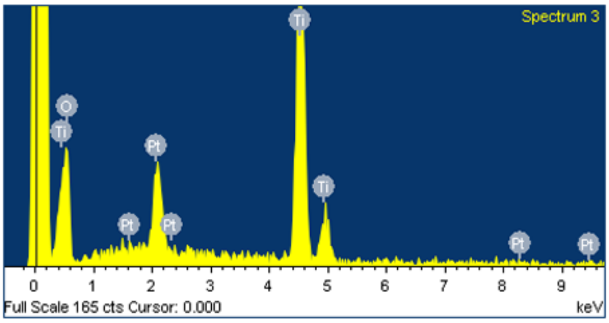

Figure S1: EDX spectrum of Pt@TiO_2 nanoparticles. The quantitative analysis shows that the molar ratio Pt/TiO_2 is 6.7%, which is quite close to the standard value of 5%.

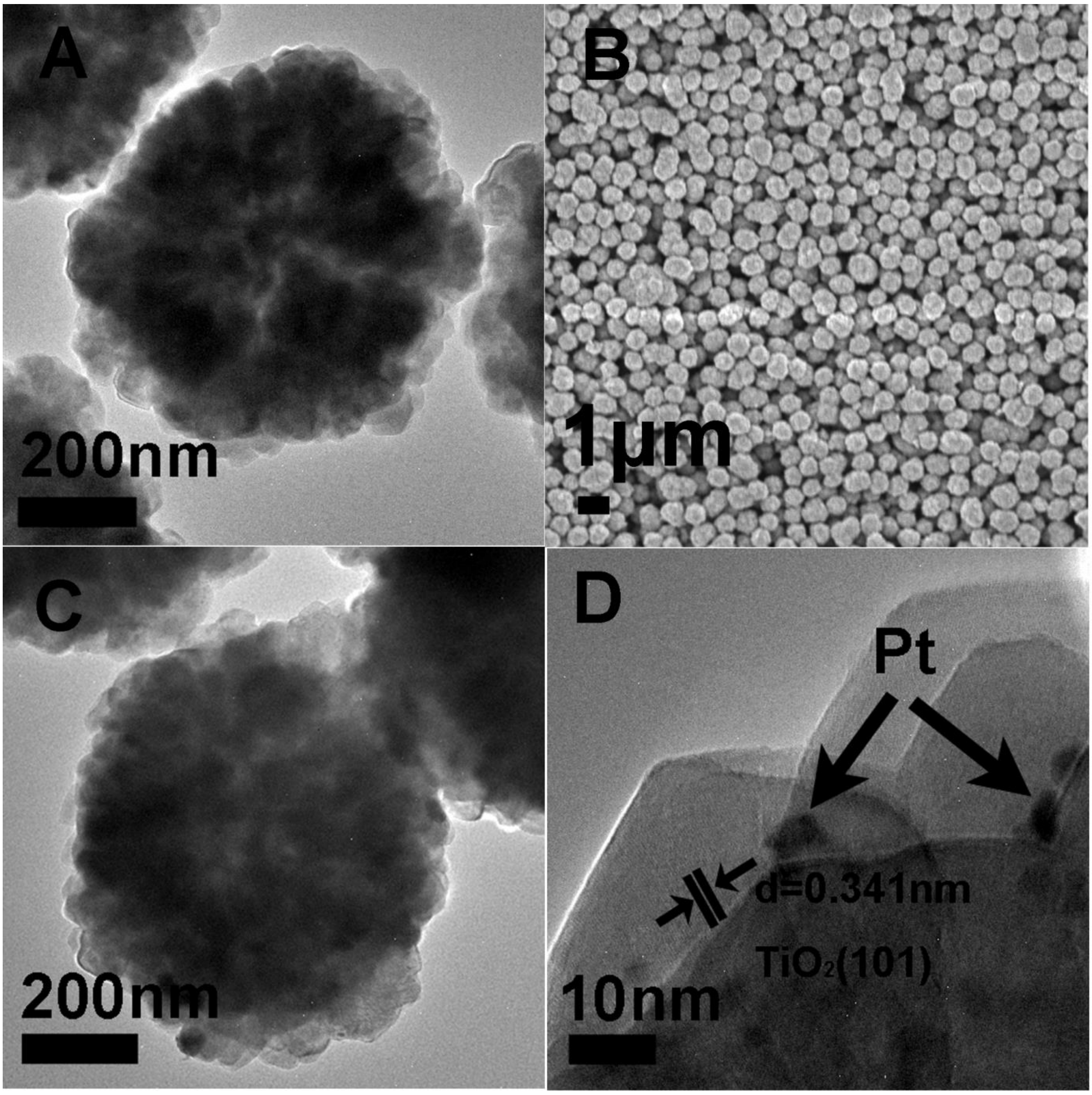

Figure S2: The TEM and SEM images of the Pt/TiO_2 photocatalyst. (A) TEM image of bare TiO_2, (B) SEM image of Pt/TiO_2, (C) TEM image of Pt/TiO_2, (D) HRTEM image of Pt/TiO_2.

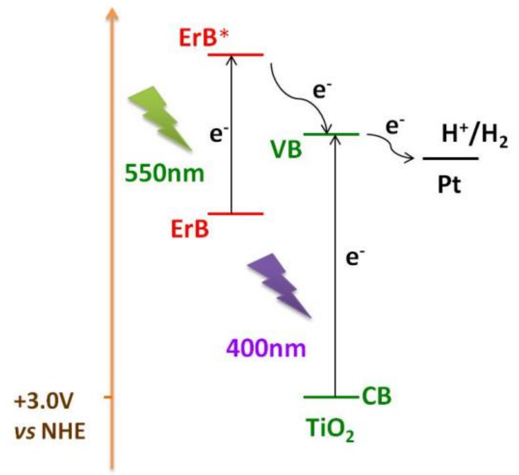

Figure S3: Energy diagram of ErB-sensitized Pt@TiO_2 for the generation of H_2 from proton reduction.

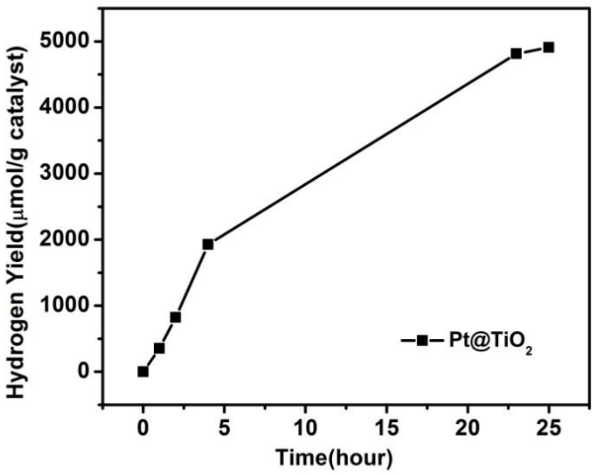

Figure S4: Long term test of H_2 evolution by ErB-Pt@TiO_2 under irradiation at 550 ± 20 nm.
